# Organoid Brain‐Machine‐Interface Devices for Central Nervous System Repair

**DOI:** 10.1002/advs.75444

**Published:** 2026-04-27

**Authors:** Yantao Xing, Yang Yang, Zichen Hong, Chunhui Tian, Huiyu Chu, Susanne Clara Prokop, Hongwei Cai, Mingxia Gu, Jason Tchieu, Ken Mackie, Feng Guo

**Affiliations:** ^1^ Department of Intelligent Systems Engineering Indiana University Bloomington Bloomington Indiana USA; ^2^ Department of Medicine Harvard Medical School Brigham and Women's Hospital Boston Massachusetts USA; ^3^ Gill Institute for Neuroscience Science Department of Psychological and Brain Sciences Indiana University Bloomington Bloomington Indiana USA; ^4^ Department of Anesthesiology and Perioperative Medicine David Geffen School of Medicine Eli and Edythe Broad Center for Regenerative Medicine and Stem Cell Biology University of California Los Angeles California USA; ^5^ Center For Stem Cell and Organoid Medicine (CuSTOM) Division of Developmental Biology Division of Pulmonary Biology Cincinnati Children's Hospital Medical Center Cincinnati Ohio USA; ^6^ University of Cincinnati School of Medicine Cincinnati Ohio USA

**Keywords:** brain‐machine‐interfaces (BMI), central nervous system (CNS), neural devices, organoids

## Abstract

Central nervous system (CNS) repair and regeneration suffer from tremendous clinical challenges due to current limitations in replacing lost neural tissues and restoring long‐term neural circuits. Neural organoids, 3D lab‐cultured neural tissues derived from stem cells, can recapitulate key cellular, structural, and physiological features of the human CNS, showing promising potential for neural regeneration. Here, we envision organoid brain‐machine‐interface (Organoid‐BMI) devices as a new kind of neuroelectrical interface for CNS repair. The Organoid‐BMI devices employ neural organoids and bioelectrodes as biohybrid bidirectional communication pathways to connect the human CNS and the external world. Acting as a biologically compatible intermediate, this approach may facilitate structural incorporation and functional alignment with host neural circuits for addressing persistent challenges of CNS repair including graft‐host mismatch and long‐term circuit stability. Through implementing adaptive and closed‐loop strategies, this approach can modulate interaction and functional communication with the host for promoting CNS circuit remodeling and functional recovery. Together, this innovative technology may open new avenues for personalized regenerative medicine.

## Graphical abstract

We envision organoid brain‐machine‐interface (Organoid‐BMI) devices as new biohybrid bidirectional communication pathways to connect the human CNS and the external world for personalized CNS repair and regeneration.

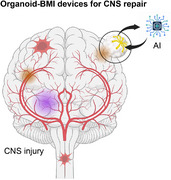

## 1 Introduction

Disorders and injuries of the central nervous system (CNS), including stroke, brain and spinal cord injury, remain a major clinical challenge [1, 2, 3, 4]. CNS injury may disrupt motor, sensory, cognitive, and autonomic function of an individual patient, and can even lead to a loss of independence [5]. For example, stroke often causes bladder problems and speech or visual deficits [6], while brain and spinal cord injury frequently leads to persistent cognitive problems and behavioral changes [7]. Despite reducing secondary damage and supporting adaptation, current clinical treatments (e.g., surgery, medication, and rehabilitation [7]) still cannot rebuild neural tissues or restore native circuitry [8]. There is an urgent and unmet need to develop novel therapies and treatment strategies for promoting neural repair and functional recovery [9]. Recently, brain‐machine interfaces (BMIs) have attracted tremendous attention for CNS repair. This approach may bypass damaged pathways using external devices to record and convert neural activity into control signals [10]. For example, BMIs may employ robotic limbs or computer interfaces to restore movement or communication [11, 12, 13, 14, 15]. Compared with conventional assistive technologies, BMIs can improve current rehabilitation protocols due to their better control precision and real‐time adaptability. However, challenges remain for clinical translation of BMIs. Non‐invasive BMI systems have limited spatial resolution and are susceptible to interference, while invasive BMI approaches provide high precision but suffer from significant long‐term stability and safety issues. Moreover, the efficacy of BMI technologies in treating CNS injuries varies across individuals, as it depends on patients’ cognitive status, rehabilitation goals, and environmental conditions [16, 17, 18, 19].

Neural organoids, such as brain organoids and spinal cord organoids, can recapitulate key molecular, cellular, structural, and physiological features of the human CNS [20, 21, 22, 23, 24, 25, 26, 27], and these 3D neural tissues derived from stem cells hold remarkable potential for basic neuroscience research and translational regenerative medicine [28, 29, 30]. So far, the neural organoids have been transplanted as living grafts, and preclinical studies also suggest that transplanted organoids can survive, vascularize, functionally couple with the host, and even enhance motor, sensory, and cognitive performance [31, 32, 33]. Compared with cell suspensions and scaffold‐based transplants, organoid grafts may provide better functional integration based on their more physiologically relevant structures with pre‐organized cell layers and intrinsic network activity. Neural organoid transplantation has emerged as a promising experimental strategy for CNS repair, since the transplanted organoids may promote CNS repair through multiple pathways: (i) cell replacement: neurons and glial cells of transplanted organoids can integrate into host neural circuits [34]; (ii) circuit reconstruction: transplanted organoids can form synaptic connections with host neurons to reestablish functional pathways [35]; and (iii) neurotrophic support: transplanted organoids may act as trophic cues and thus modulate local microenvironment and enhance endogenous repair of the host [36], and local neurotrophic factor expression and release can be regulated by the neural activity through electrical stimulation or interface‐mediated control [37].

Despite tremendous potential, significant challenges remain for CNS repair using neural organoids. The current neural organoids are suffering from high heterogeneity, poor penetration of oxygen and nutrients, variable reproducibility, random synaptic connectivity with host neural circuits, and risks such as immune rejection, abnormal growth, or tumorigenicity [32, 38]. Moreover, the long‐term stability, functional maturation, and effective interaction remain largely understudied after transplanting these organoid grafts into the adult CNS. These call for integrative strategies that can maximize functional recovery after CNS injury, potentially combining neuromodulation or BMI technologies. Thus, we envision organoid‐BMI devices that use neural organoids and electrodes as biohybrid bidirectional communication pathways to connect the human CNS and the external world with adaptive closed‐loop capability. In this approach, organoids could act both as repair units after transplantation and as active sensing‐processing hubs that bidirectionally interface with external devices and rehabilitation systems. This integration may restore lost function and enhance neuroplasticity, offering a synergistic path toward more complete CNS recovery. Here, we outline the concept, technological needs, translational potential, and challenges of Organoid‐BMI devices.

## 2 Concept of Organoid‐BMI Devices

**FIGURE 1:**
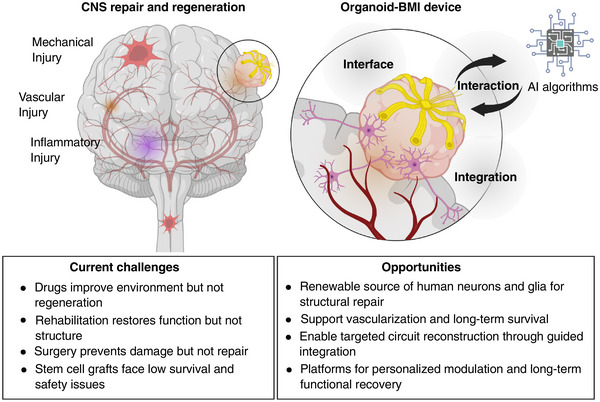
Organoid‐BMI devices for CNS repair. Neural organoids integrated with electronic interfaces provide new opportunities for CNS repair. Organoids can serve as sources of human neurons and glia for promoting structural repair, vascularization, and long‐term survival in host CNS tissues, and enable guided circuit reconstruction through targeted integration. Combined with the closed‐loop regulation and the AI‐assisted control, Organoid‐BMI devices hold promise for personalized neuromodulation and long‐term functional recovery. Elements created with BioRender.com.

Organoid‐BMI devices are a biohybrid approach that integrates neural organoids and bioelectronics with closed‐loop modulation for connecting the human CNS and the external world (Figure 1). Unlike conventional BMIs that rely on direct coupling between bioelectronics and brain tissues, Organoid‐BMI devices introduce neural organoids as a living and adaptive intermediary layer. In this configuration, the organoid is neither a replacement for the brain nor a passive graft, but a functional component of the interface itself, capable of autonomous network activity, learning, and modulation. According to the protocols, neural organoids may contain various CNS cells such as neurons, neuron progenitors, astrocytes, and other supporting cells. They are not simply passive grafts but act as living neural substrates with spontaneous and evoked network activity. Integrating with flexible electrodes (optical approaches, or microfluidic components, if appropriate), organoid activity is recorded and modulated in real time by a regulation/feedback layer that delivers precisely parameterized stimulation or sensory surrogate inputs. Together, the biological, electronic, and control modules can form a bidirectional, closed‐loop interface to connect the human CNS and the external world.

The controllability and predictability of Organoid‐BMI devices depend strongly on the type and internal organization of the organoids employed. In practice, initial Organoid‐BMI device transplantation may preferentially employ organoids with relatively constrained cellular compositions or guided patterning, such as those enriched for predominantly excitatory or inhibitory neuronal populations, as these systems are more likely to exhibit stereotyped and interpretable responses to stimulation. By contrast, organoids with more complex and heterogeneous cellular compositions can display high‐dimensional, non‐linear dynamics [39], making stimulus‐response relationships and corresponding release criteria difficult to define a priori. In addition, the structural features of neural organoids, including laminar patterning, cytoarchitecture, and regional identity, vary substantially across differentiation protocols, developmental stages, and batches, and not all organoids exhibit clear laminar organization [40]. Within this design space, more complex organoids are better suited for later‐stage or application‐driven studies, whereas the purpose of the BMI is not to rely on a fixed or uniform organoid architecture, but to provide an adaptive, closed‐loop framework that infers network state and modulates activity in a data‐driven manner, enabling individualized calibration of stimulation and control strategies across diverse organoid types.

Organoid‐BMI devices can be designed along two complementary pathways, in vitro pathway and in vivo pathway. For the former, Organoid‐BMI devices aim to (i) assemble mature organoids toward electrophysiological competence; (ii) record network states with high‐density flexible bioelectrodes and stimulate with high spatiotemporal precision; and (iii) form a closed‐loop system that can track network state and deliver individualized stimulation protocols to shape connectivity and plasticity. This platform will be designed for rapid iteration of signal‐interpretation and control strategies, stimulation‐response studies, and preclinical screening of rehabilitation‐relevant training regimens before moving in vivo. On the other hand, for in vivo pathway, Organoid‐BMI devices extend the concept to transplantation. Here, organoids are pre‐matured and pre‐interfaced with compliant microelectrode meshes or conformable cuffs to secure robust coupling at implantation. The transplant procedure is coordinated with stereotactic delivery and microsurgical workflows to minimize shear and hypoxia while preserving orientation. After transplantation, interface integration prioritizes the electrode geometry and placement, mechanical fixation compatible with tissue micromotion, and strategies for long‐term stability (hydrogel encapsulation, substrate softening, impedance management). The feedback layer then links the device and host: recorded activity guides adaptive stimulation of the organoid, adjacent host cortex, or both, with the aim of biasing Hebbian/anti‐Hebbian plasticity, stabilizing desirable oscillatory regimes, and progressively recruiting host circuits into behaviorally useful pathways [41].

Thus, Organoid‐BMI devices reframe organoid transplantation from a static cell‐replacement strategy into a dynamic, co‐adaptive interface. The organoid supplies cellular diversity and retains developmental programs that support plasticity and responsiveness to host cues; bioelectronics provide high‐fidelity input‐output with mechanics matched to tissue; and the control layer enables task‐guided, state‐dependent modulation. In combination, these elements confer two roles within a single device: (i) a biological repair unit for reconstructing microcircuitry; and (ii) an active modulatory hub for amplifying activity patterns with closed‐loop feedback over clinically relevant timescales. Thus, Organoid‐BMI devices may provide adjustable, measurable, and responsive neural systems that can evolve with the patient's recovery.

## 3 Opportunities for CNS Repair

Organoid‐BMI devices may bring new opportunities for CNS repair. First, Organoid‐BMI devices provide a unique source of human neurons and glia for structural repair. Unlike clinical methods that mainly improve the microenvironment or compensate for function (Table 1), and unlike current BMIs that cannot provide cellular replacement [42, 43, 44, 45, 46, 47], organoid‐based strategies directly deliver new human neural cells. What distinguishes Organoid‐BMI devices from simple organoid transplantation is that grafts can be pre‐characterized in vitro using electrophysiological and molecular “release criteria” to confirm functional integrity, laminar organization, and excitability before implantation (Table 1). Once in vivo, real‐time electrophysiological recordings can verify graft activity and support adaptive regulation, transforming cell replacement from a probabilistic intervention into a guided process. Second, Organoid‐BMI devices may support long‐term survival and functional neural network integrity by integrating perfusion, monitoring, and feedback control units. This approach can leverage biosensors for tracking nutrient supply/metabolic dynamics, perfusable scaffolds and microfluidic systems for modulating perfusion of nutrients and oxygen, and feedback control strategies for establishing conditions more favorable for graft survival [48, 49, 50]. Third, Organoid‐BMI devices enable targeted circuit reconstruction through guided integration. Conventional organoid transplantation carries the risk of random wiring, while BMIs only bypass damaged pathways. Organoid‐BMI devices can guide axonal growth and synapse formation to selectively strengthen target graft‐host connections by leveraging activity‐dependent stimulation and precision grafting. This process can be validated through connectivity metrics (e.g., directional coherence and cross‐frequency coupling) and preclinical connectivity mapping (e.g., viral tracing). Finally, Organoid‐BMI devices offer a platform for personalized modulation and long‐term functional recovery. Different from the open‐ or semi‐closed‐loop systems (e.g., deep brain stimulation or transcranial magnetic stimulation) with infrequent parameter updates, Organoid‐BMI devices use continuous readouts from both host and organoid graft to adaptively optimize stimulation strategies. This allows the system to capture transient plasticity windows during rehabilitation and consolidate them in real time. Through the integration of AI, biomarkers can be extracted and aligned with behavioral outcomes. Thus, Organoid‐BMI devices may enable evidence‐based rehabilitation trajectories of an individual, which is hardly achievable using traditional methods.

**TABLE 1:** Comparison of Current Approaches for CNS Repair.

Approach	Main Strategy	Advantages	Limitations
Clinical methods (surgery, drugs, rehabilitation, stem cell therapy)	Surgery reduces damage; drugs improve microenvironment; rehab aids compensation; stem cells supply new cells	Established clinical use; safe; stem cells replace dead brain cells	Cannot rebuild large lesions; drugs/rehab symptomatic only; stem cells immature, poor vascularization, low survival, immune risk
Brain‐machine interface (BMI)	Electrodes for brain‐device communication	Functional compensation; clinical applications (prosthetics, communication)	No tissue replacement; limited applicability to large‐scale defects
Brain organoid transplantation	hiPSC/hESC‐derived organoids transplanted to the lesion	Provides neurons/glia; partial local repair; vascularization/synapses in models	Immature; poor vascularization; random wiring; unstable long‐term
Organoid BMI devices	Organoid graft + neural interface + closed‐loop control	Combines cellular replacement with adaptive modulation; real‐time monitoring	Early stage; maturity, heterogeneity, safety, multimodal challenges

## 4 Organoid‐Electrode Interfaces

To function reliably over extended culture duration, organoid‐electrode interfaces must meet several key criteria, including biocompatibility, long‐term stability, low‐impedance electrical coupling, and mechanical compliance to minimize tissue damage. In this section, we discuss interfacing paradigms, material selection, interface design, and structural strategies (Table 2).

**TABLE 2:** Comparison of organoid‐electrode interfacing strategies.

Interface type	Structure	Materials	Signal depth	Advantages	Limitations
Surface‐conformal	Folding shells, wrapping meshes	PI, SU‐8, elastomers,Ga‐based LM, Au/Pt	Surface	Noninvasive; high SNR at surface; culture‐friendly	Limited depth; core signal attenuation
Biohybrid	Embedded meshes (co‐development)	Flexible nanoelectronics, polymers	Surface‐intermediate	Developmental integration; long‐term stability	Early‐stage integration; fabrication complexity
Penetrating (rigid/semi‐flexible)	Microneedles, multishank probes	Si, PI shafts, Au/Pt	Deep	High resolution; internal access	Tissue damage; mechanical mismatch
Penetrating (soft/adaptive)	Deformable probes	Ga‐based liquid metal, elastomers	Deep	Reduced mismatch; improved chronic stability	Emerging; limited long‐term data

### 4.1 Surface‐Conformal Interfaces

**FIGURE 2:**
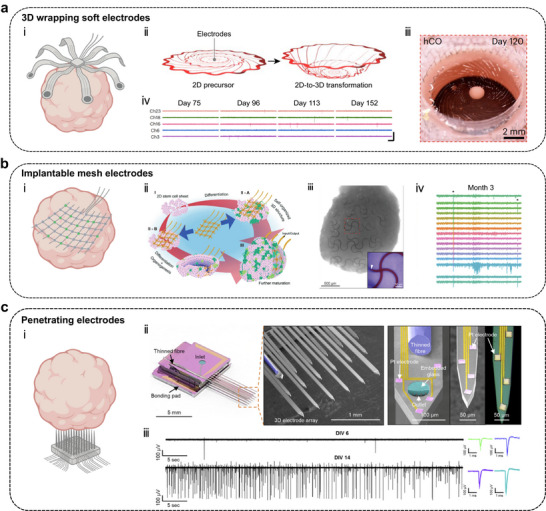
Organoid‐electrode interface strategies. (a) 3D wrapping soft electrodes: A soft design allows a 2D precursor to fold into a 3D wrapping electrode array that conforms around brain organoids, enabling long‐term electrophysiological monitoring. Adapted from Ref. [56]. with permission (2024 Nature Portfolio). Elements created with BioRender.com. (b) Implantable mesh electrodes: A flexible mesh‐like electrode sheet integrates with organoids during development for intimate bioelectronic coupling and stable recording of neural activity. Adapted from Ref. [60]. with permission (2019 American Chemical Society) and from Refs. [59, 61]. under CC BY 4.0. Elements created with BioRender.com. (c) Penetrating electrodes: High‐density penetrating microelectrodes enable intratissue access to deep organoid regions for capturing rich spiking and network dynamics with high temporal and spatial resolution. Adapted from Ref. [64]. under CC BY 4.0. Elements created with BioRender.com.

Surface‐conformal interfacing strategies aim for noninvasive recording of superficial neural network activity by tightly wrapping flexible electrode arrays around the curved surfaces of brain organoids (Figure 2a,i). This approach has significantly expanded the repertoire of 3D encapsulating interface designs. For instance, SU8‐based self‐folding shell electrodes were developed to enable 3D encapsulation of organoids, resulting in markedly improved signal‐to‐noise ratio (SNR) [51]. A micropatterned multielectrode shell was introduced to enable 3D capture of spatiotemporal activity from living cells [52]. Self‐rolling techniques were employed to construct “organ‐on‐a‐chip” platforms, enabling electrophysiological investigation and monitoring of complex signal transduction within 3D cellular assemblies [53, 54]. A hydrogel‐actuated “e‐flower” electrode was further developed, whose blooming morphology enabled dynamic encapsulation of brain spheroids [55]. Principles of Japanese kirigami were leveraged to fabricate stretchable and highly conformal electronic devices for long‐term monitoring of organoids and assembloids [56] (Figure 2a,ii‐iv). Mechanics‐driven self‐assembly was utilized to design 3D flexible electrode interfaces that gently envelop spheroidal organoids, enabling high‐resolution heating, stimulation, and monitoring [57]. Collectively, these innovations have advanced noninvasive interfacing and functional interrogation of organoids, as exemplified by the successful application of highly stretchable 3D microelectrode arrays for functional assessment of midbrain organoids and cardiac spheroids [58].

The advantages of 3D shell‐like conformal interfaces are evident. Compared with traditional planar electrodes, shell electrodes maximize the physical contact area with the curved surface of organoids, thereby substantially enhancing recording performance. Empirical data demonstrate that 3D shell electrodes detect far more neuronal spikes than their 2D counterparts (7785 vs. 2025), with a 42% increase in median SNR [51]. Their noninvasive nature not only preserves tissue integrity but also simplifies culture handling. Despite these advantages, challenges remain. For example, signals are predominantly captured from surface‐layer neurons but not deeper internal neurons, limiting the assessment of the full network dynamics. Moreover, although flexible and adjustable, achieving perfect conformity to irregular organoid morphologies and maintaining stable recordings under cyclic deformations during long‐term culture still pose major technical challenges.

### 4.2 Biohybrid Interfaces

Biohybrid interfacing represents a deeper level of integration, shifting the focus from simply “connecting” tissues to embedding electronic devices as integral components of the tissue architecture itself [59] (Figure 2b,i). Several innovative strategies exemplify this paradigm. Most notably, the concept of cyborg organoids was introduced, in which nanoelectronic devices were incorporated at early stages of organogenesis, enabling the co‐development of electronics and tissue to achieve structural‐level integration [60] (Figure 2b,iii‐iv). Building on this concept, chronic single‐cell‐level recording of developing organoids over several months was achieved [61] (Figure 2b,ii). Mesh‐like microelectrode arrays were further demonstrated to function both as structural scaffolds for organoids and as internal platforms for electrophysiological monitoring [62]. The development of biohybrid interfaces represents a shift from merely interfacing with tissue toward structural integration within living systems. When organoids incorporate electronic meshes during development, the device transitions from an exogenous implant to a stably embedded and developmentally integrated interface. Current studies primarily demonstrate long‐term stability and passive electrophysiological recording; however, this level of developmental and mechanical integration establishes key prerequisites for future closed‐loop modulation. Notably, such biohybrid integration has been shown to persist stably within organoids for up to one year [62], while reducing chronic inflammation and foreign‐body responses [63]. Despite these promising advances, several challenges remain for fabricating and transplanting Organoid‐BMI devices for CNS repair.

### 4.3 Penetrating Interfaces

To fully explore the potential of 3D neural organoids, it is critical to develop technologies for volumetric recording and stimulation of neurons throughout the whole organoid. Current surface‐based approaches lack the penetration depth, while conventional single‐electrode probes do not provide network‐level analysis. Penetrating interfaces address these limitations by inserting microneedle or micropillar arrays directly into organoids, thereby enabling high‐fidelity recording of deep neural signals (Figure 2c,i). For example, a multilayer stacking and bonding strategy was employed to construct 3D high‐density electrodes that not only provide high‐resolution recordings but also integrate optical stimulation and drug delivery functionalities, offering a multimodal platform for probing neural circuit dynamics [64] (Figure 2c,ii‐iii). Pushing spatial resolution further, high‐density, independently addressable nanowire arrays are used to achieve intracellular recordings from neurons, delivering exceptional 3D spatiotemporal resolution and SNR [65]. In response to this trade‐off, recent work has introduced soft, shape‐adaptive penetrating neural probes designed to reduce mechanical mismatch while preserving access to deep 3D activity [66, 67, 68]. Unlike rigid or semi‐flexible probes, these interfaces are engineered to deform or reconfigure after insertion for better accommodation of tissue motion and growth. For example, Ga‐based liquid metal‐enabled 3D microelectrode arrays, developed by the Park group, have demonstrated high mechanical compliance, resilience under large strain, and stable volumetric electrical recording, highlighting their potential for long‐term organoid integration and translational applications [69, 70].

However, these advanced performance gains come with a major tradeoff. Invasive neural interfaces can capture high‐quality signals, but they also disrupt the surrounding biology. When electrodes penetrate tissue, they cause immediate damage and trigger long‐term immune responses, causing scar formation around the electrodes, further weakening signal quality. In growing and remodeling organoids, fixed electrodes can also create ongoing mechanical stress or become misaligned, further reducing recording stability. To mitigate such adverse effects, flexible probes with hinge‐like regions at their bases were developed to deform plastically while maintaining vertical alignment [71]. This strategy minimizes invasiveness by allowing pre‐positioned probes to be embedded within a cast of 3D cell‐matrix mixtures, enabling neural networks to develop in situ around the probe and thereby avoiding insertion‐related injury to preformed tissue. Using this approach, long‐term co‐culture of human iPSC‐derived neurons and astrocytes was successfully maintained. With ongoing advances, the utility of penetrating interfaces now extends beyond passive high‐fidelity recording of internal organoid activity to integration within more complex systems. High‐density silicon probes are used to perform extracellular recordings from long‐term human brain organoids, providing evidence that brain organoids not only recapitulate aspects of structural development but also spontaneously generate active neural networks with complex dynamics [72, 73].

### 4.4 Material Selection for Organoid‐Electrode Interfaces

Mechanical mismatches remain as fundamental issues for conformal contact and long‐term stability of organoid‐electrode interfaces. The large disparity in Young's modulus between rigid electrodes and soft brain tissue is a major cause of chronic inflammation and device failure. For example, the Young's modulus of brain tissue typically lies in the kPa range, whereas conventional silicon‐based electrodes exhibit moduli in the tens to hundreds of GPa, resulting in a mismatch spanning several orders of magnitude [74]. Consequently, the use of flexible substrates that match the mechanical properties of neural tissue has become a cornerstone of interface design, with biocompatible polymers serving as the structural backbone of these devices. Polyimide (PI), owing to its biocompatibility, chemical stability, and compatibility with standard micro/nanofabrication processes, is widely adopted as a substrate and encapsulation layer in stretchable 3D electrodes and 3D multifunctional mesoscale frameworks [57, 58]. SU‐8, a commonly used negative photoresist, has been widely employed for its excellent microfabrication compatibility and processing versatility in stretchable mesh nanoelectronics [61]. Although SU‐8 is intrinsically stiff, effective mechanical compliance in these systems is achieved primarily through reduced thickness and structural design rather than the bulk material properties of SU‐8 itself. Elastomers such as thermoplastic polyurethane (TPU) and polyurethane (PU), with their superior elasticity, have also been employed to fabricate liquid‐metal‐based devices, enabling extreme stretchability [75].

While flexible materials alleviate mechanical mismatch, achieving high‐quality electrophysiological signals additionally requires overcoming challenges at the electrical interface between tissue and electrodes. The conductive layer critically determines the efficiency and fidelity of signal transmission, making material choice equally essential. For example, surface‐modified electrodes such as Pt black or PEDOT:PSS typically reduce impedance by one to two orders of magnitude compared with bare metal electrodes, thereby improving recording sensitivity. Noble metals such as gold (Au), platinum (Pt), and titanium (Ti) are commonly used for electrodes and interconnects due to their high conductivity and chemical inertness. Recently, innovative liquid‐metal‐polymer composites, in which micro/nanoparticles of gallium‐indium alloys are dispersed within elastomeric matrices (e.g., TPU/PU), have been used as new electrode materials, which maintain continuity under deformation and offer up to 500% stretchability while retaining conductivity and mechanical compliance [75].

Beyond material selection, a central challenge for noninvasive interfaces arises from the weak contact pressure between floating organoids and electrodes in culture media. This loose coupling results in enlarged cell‐electrode gaps, thereby increasing impedance, reducing SNR, and distorting signals. This relationship highlights that effective interface performance relies on the combination of mechanical contact, interfacial impedance, and electrochemical properties, rather than on material composition alone. To address this issue, over‐electrodeposition of the conductive polymer PEDOT:PSS was employed to fabricate microelectrodes that physically protrude above the device's insulating layer. This design enhanced mechanical contact pressure and, as validated by Finite Element Method simulations, led to a pronounced improvement in SNR (SNR > 9 for midbrain organoids and > 17 for cardiac spheroids) [58]. Another widely used strategy involves electrode surface modification with high‐surface‐area, low‐impedance materials. For example, Pt black deposition significantly increases effective electrode surface area and promotes charge transfer [57], while PEDOT coatings reduce impedance and improve long‐term stability [75], thereby enhancing signal fidelity without altering macroscopic contact pressure. In addition to bulk materials and conductive coatings, surface coating also plays a critical role in determining the long‐term organoid‐electrode performance. Anti‐fouling and anti‐inflammatory surface modifications, such as hydrophilic polymer brushes, zwitterionic coatings, or bioactive molecule functionalization, have shown enhanced chronic stability and biocompatibility by reducing protein adsorption, attenuating glial activation, and/or mitigating tissue damage at neural interfaces [76, 77].

In contrast to noninvasive flexible interfaces, penetrating probes physically, thereby offering a unique window into neuronal activity within deeper layers. These probes are typically fabricated using standard semiconductor industry technologies by constructing high‐precision and high‐density electrode arrays on silicon substrates. Moreover, larger multi‐shank probes can be fabricated on shaft materials such as polyimide to provide limited flexibility while retaining sufficient stiffness for insertion [71]. The fabrication of penetrating probes has advanced beyond the constraints of traditional planar processes. An innovative method was developed to produce high‐density vertical nanowire arrays [65]. This approach employed an all‐solid‐state wafer bonding integration scheme, wherein nickel silicidation was used to fuse silicon wafers with sapphire substrates pre‐patterned with electrode interconnects. This breakthrough enabled, for the first time, independent electrical addressing of individual nanowires within high‐density vertical arrays—an achievement unattainable with conventional techniques. Another representative advance was demonstrated with the introduction of stacked 3D multi‐shank arrays [64]. A 3D electrode array was built by stacking and bonding three separate 2D multi‐shank devices, allowing the probe to span entire organoids. This modular approach offers a scalable way to create larger recording volumes. Together, advances in materials and microfabrication form the technical basis for the high performance of penetrating probes, shaping their recording capacity and resolution.

### 4.5 Structural Strategies for Stable Organoid‐Electrode Interfaces

In addition to material innovations, researchers have developed diverse structural strategies to address the dynamic challenges of living organoid culture and to ensure long‐term interface stability. Soft mesh buffering and support strategy focus on designing flexible, porous 3D electronic scaffolds with tissue‐like mechanical properties that physically accommodate or envelop organoids. The goal is to provide a stable environment for culture and recording—particularly under suspension culture conditions—while minimizing interference with self‐organization and developmental processes. Representative examples include *kirigami* electronics [56], mechanics‐guided self‐assembling flexible electrodes [57], and liquid‐metal‐polymer conductor based mesh interfaces [75]. These approaches physically support organoids in suspension, offering buffering and fixation while enabling long‐term, noninvasive electrophysiological recording. Unlike post hoc interfacing strategies, pre‐integration introduces a disruptive concept: incorporating electronic devices into stem cells or progenitors during organoid development. Leveraging intrinsic morphogenetic forces, the tissue self‐organizes to seamlessly embed electronics within its 3D structure, as exemplified by *cyborg organoids* [60]. This approach circumvents acute implantation injury and the associated inflammatory responses by reframing electrodes as part of the developmental environment rather than foreign implants. Such seamless incorporation achieves greater long‐term stability and provides an unprecedented advantage for capturing the origins of neural activity. Notably, this strategy has enabled the detection of the very first action potentials from individual neurons during early development and the tracking of their maturation trajectories over several months.

For many practical applications, particularly when using commercially available or conventionally cultured organoids whose early developmental protocols cannot be modified, pre‐integration is not feasible. Shape‐adaptive flexible electronics address this limitation by employing devices fabricated in planar form that undergo controlled 2D‐to‐3D transformations, conformally encapsulating preformed organoids. For example, bilayer polymer membranes with distinct crosslinking degrees engineered through solvent‐driven folding into shell‐ or cap‐like structures to conformally wrap organoids [51]. These plug‐and‐play, standardized, and user‐friendly 3D electrode platforms substantially lower the barrier for implementing 3D electrophysiology in organoid research, with promising applications in drug screening and personalized medicine. Stable, long‐term electrophysiological studies of 3D organoids require strategies to minimize relative motion between tissue and electrodes, thereby reducing motion artifacts and ensuring high‐SNR, high‐fidelity signal acquisition. This is particularly crucial for penetrating interfaces, where reliable cell‐electrode coupling is essential. A simple physical anchoring approach is employed by placing organoids on a small grid of spikes to immobilize them during probe recordings [72]. By contrast, a more integrated dual‐function design with vertically aligned nanowire arrays serves both as independently addressable electrodes and as microstructural supports to stabilize organoids [65]. During culture, neurons actively grow around and “wrap” these nanowires, forming tight and stable bioelectronic interfaces.

## 5 Integration Mechanisms Between Organoids and Hosts

After achieving stable signal exchange at the organoid‐electrode interface, integration between the organoid and the host becomes the core step for realizing long‐term neurorepair and functional reconstruction. Integration not only determines whether the organoid can survive in the host, but also directly affects its ability to form effective input‐output circuits and ultimately translate into observable behavioral improvement and enhancement of network function [78, 79, 80]. Evidence from animal and preclinical studies suggests that this process spans three major levels: structural integration (e.g., vascularization, modulation of glial responses, and tissue matching); cellular integration (e.g., bidirectional axonal growth, synaptogenesis, and projection specificity); and functional integration (e.g., encompassing synchronization of electrophysiological activity, responsiveness to external stimulation, and participation in behavioral tasks). Representative work from the Pasca group has provided in vivo evidence for multilevel integration of transplanted human cortical organoids [78]. At present, vascularization and infiltration by supportive host glia at the structural level have been confirmed in multiple animal experiments. At the connectivity level, there is evidence that organoids receive host inputs and send projections outward. At the functional level, organoids show stimulus‐evoked activity and early signs of behavioral improvement. Nevertheless, major challenges remain in achieving full restoration of complex, task‐level functions.

### 5.1 Structural Integration

**FIGURE 3:**
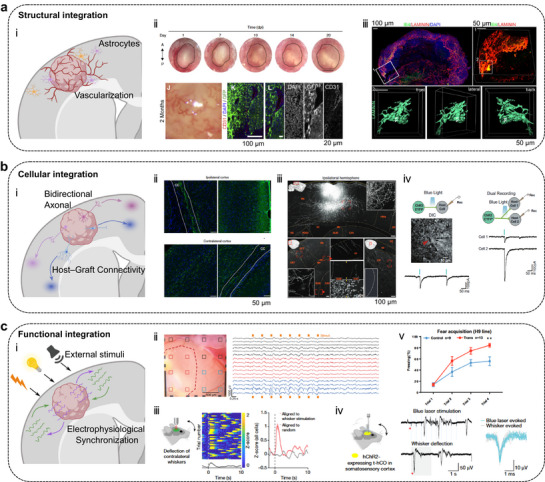
Multilevel integration of transplanted organoids with host neural tissues. (a) Structural integration. Transplanted organoids exhibit host vascular network fusion, where host‐derived vessels infiltrate the graft to provide oxygen and nutrients, reducing hypoxia and apoptosis. Pre‐vascularization and integration with host vasculature further improve graft survival and metabolic stability. Adapted from Refs. [34, 81]. with permission (2018 Nature Portfolio, 2023 Cell Press) and from Ref. [83]. under CC BY 4.0. Elements created with BioRender.com. (b) Cellular integration. Organoid‐derived axons extend into the host cortex and corpus callosum, supporting interhemispheric connectivity. Viral tracing and optogenetic experiments reveal projection specificity, indicating that graft neurons target appropriate host regions. Bidirectional synaptic communication is demonstrated by recordings showing signal transmission from grafted cells to host circuits. Adapted from Refs. [34, 85]. with permission (2018 Nature Portfolio, 2016 Nature Portfolio) and from Ref. [33]. under CC BY 4.0. Elements created with BioRender.com. (c) Functional integration. Organoids exhibit spontaneous network activity in vivo and respond to host‐derived sensory stimuli, such as whisker deflection or optogenetic input, indicating functional coupling. Behavioral assays, including fear conditioning and motor‐related tasks, demonstrate that graft integration translates into observable functional recovery in host animals. Adapted from Refs. [33, 79, 89]. under CC BY 4.0. Elements created with BioRender.com.

Following transplantation, organoids are typically perfused by the host vasculature and become vascularized, thereby ensuring sustained delivery of nutrients and oxygen, which is an essential prerequisite for subsequent integration and functional recovery (Figure 3a,i). For example, in immunodeficient mice, host vessels have been observed invading human PSC‐derived cerebral organoids and forming functional vascular networks, resulting in superior survival and growth relative to in vitro culture [34]. Consistently, transplantation of ∼80‐day human cortical organoids into adult rat visual cortex lesions resulted in enlarged grafts with abundant host‐derived vasculature three months after transplantation [81, 82]. By rapidly reducing intra‐graft hypoxia and necrosis, vascular integration provides a more stable metabolic milieu and supports further organoid maturation. Once vascularization establishes the basis for survival, glia‐graft‐host interactions become an important determinant for the quality of integration [34, 81, 83] (Figure 3a, ii‐iii). On the one hand, organoids undergo progressive gliogenesis in vivo, generating cells with astrocytic features; in mouse transplantation models, GFAP‐positive cells have been identified within grafts [34], alongside infiltration of host microglia that aid debris clearance and neuronal survival. On the other hand, increasing the glial content of organoids prior to transplantation has also been shown to enhance graft stability [84]. Glia‐enriched human cortical organoids generated through prolonged differentiation exhibit mature astrocytic features, including endfeet closely associated with vasculature and the formation of blood–brain barrier–like structures. These results suggest that graft‐derived glial cells can provide metabolic and structural support and facilitate the integration of organoids into the host neurovascular system. Moreover, a balanced glial response limits scar‐like isolation while promoting supportive glia, enhancing graft adaptation and functional connectivity [82, 83]. Due to immune rejection during the transplantation of human‐derived organoids into animals, most studies use immunodeficient rodent models or immunosuppression. For example, to achieve long‐term graft survival, human cortical organoids have been transplanted into neonatal athymic rats [79] and NOD‐SCID mice [32, 34], which lack functional T and B cells. In these models, organoids survived for months, matured into diverse neuronal subtypes, and contributed to tissue repair without clear signs of rejection. Transplantation into neonatal or juvenile brains, where plasticity is high and scarring is limited, supports axonal growth and synapse formation [79]. Overall, with appropriate host models and timing, transplanted organoids can integrate structurally, connect with host vasculature, and function within the neural environment.

### 5.2 Cellular Integration

Experimental data demonstrate that neurons from transplanted organoids can establish functional synaptic connections with host neurons [34] (Figure 3b, i‐ii). In a visual cortex injury model of adult rats, monosynaptic rabies‐based trans‐synaptic tracers injected into the retina were shown to propagate along defined visual pathways into organoid grafts, indicating direct synaptic contacts between host and grafted neurons [81, 85]. In the same study, histological data further revealed synaptic structures formed between organoid neurons and adjacent host neurons. Consistently, anatomical and tracing results also showed that transplanted human PSC‐derived cortical organoids received axonal projections from host thalamic and cortical neurons [79]. Together, these results indicate that graft neurons can be incorporated into host neural circuits and may receive and process synaptic signals from the host brain. The connectivity between organoids and host tissues is bidirectional. It has been reported that six months after transplantation, human cortical organoids not only received host neuronal projections but also extended human‐derived axons broadly across multiple rat brain regions [79]. These axons exhibited substantial length and traversed distinct anatomical areas, demonstrating the capacity of grafted neurons to form long‐range projections [85] (Figure 3b,iii). Similarly, in a stroke model, human PSC‐derived cerebral organoids transplanted into post‐stroke cortex were observed to extend long‐range axonal projections into distant host regions over several months [32]. Importantly, the graft neurons formed reciprocal synaptic connections with both local and distal host neurons. They received afferent inputs from surrounding host circuits while simultaneously sending efferent outputs to host targets [33] (Figure 3b,iv). This bidirectional axonal integration provides evidence of two‐way signal exchange between grafted organoid neurons and host circuits [86]. A critical aspect of graft‐host connectivity is the demonstration of projection specificity, indicating that organoid neurons can integrate into appropriate host circuits in a cell type‐ and topology‐dependent manner, rather than exhibiting indiscriminate outgrowth. In stroke models, organoid excitatory cortical neurons extended axons following canonical projection patterns of native cortical neurons, targeting the cortex, striatum, thalamus, and spinal cord, representing major pathways relevant to circuit reconstruction after injury [32]. Likewise, in a study of visual pathway repair, transplanted human cerebral organoid neurons integrated into the adult rat visual system and exhibited direction‐selective firing in response to specific visual stimuli, as assessed by in vivo electrophysiological recordings [81]. This functional selectivity suggests that graft neurons acquired properties characteristic of host cortical neurons. In other words, organoid grafts do not form random connections with host tissue but rather partially recapitulate developmental wiring principles, establishing circuit‐relevant connectivity. Such projection specificity may support circuit reconstruction and functional recovery [87, 88].

### 5.3 Functional Integration

After transplantation into the in vivo environment, human brain organoids exhibit more mature neural activity and network dynamics compared to those maintained in vitro (Figure 3c,i‐ii) [89]. Transplanted neurons show increased dendritic complexity, membrane excitability, and functional synapse density. For example, six months post‐transplantation, human cortical organoids exhibited synchronous rhythmic discharges (∼0.5 Hz burst activity), indicating coordinated network activity [79]. Under anesthesia, graft‐derived neuronal bursts were temporally aligned with host brain slow‐wave oscillations, suggesting that human neurons had become embedded within the broader electrophysiological patterns of the host. In vivo two‐photon imaging further revealed graft network activity that could be evoked by optogenetic stimulation and recorded electrophysiologically [79] (Figure 3c,iii). Collectively, these findings demonstrate that grafted neurons exhibit synchronized and mature firing behaviors in vivo, representing a hallmark of functional integration. Another defining feature of such integration is the ability to perceive and respond to host sensory inputs or environmental signals. In an adult mouse visual cortex transplantation model, a substantial proportion of the transplanted organoid neurons were shown to exhibit selective responses to visual stimuli, including flashing lights and grating patterns, indicating that organoid neurons received inputs from host visual pathways and participated in visual information processing [34, 89]. Similarly, transplantation of human cortical organoids into neonatal rat brains resulted in graft‐derived human neurons firing in response to host somatosensory stimulation, indicating functional incorporation into host sensory circuits [79] (Figure 3c,iv). Collectively, these findings indicate that the transplanted organoid neurons can function as both “receivers” and “processors” of host‐derived sensory signals, dynamically generating electrophysiological responses to external stimuli and cooperating with host neural systems.

The most compelling evidence of functional fusion lies in the capacity of transplanted human neural organoids to influence, and even drive, host behavior [33] (Figure 3c,v). Researchers introduced the opsin channelrhodopsin into organoid neurons and trained rats to associate light stimulation with a reward [79]. Remarkably, optogenetic activation of the transplanted organoids was sufficient to induce a learned reward‐associated behavior in host animals [79]. In other words, organoid neurons were functionally incorporated into host neuronal circuits, directly influencing behavioral outputs. Similarly, in a stroke model, mice receiving human cerebral organoid grafts showed significant improvement in sensorimotor function, including performance in the adhesive‐removal test, compared with control groups receiving dissociated cells or no graft [32]. In contrast, transplantation of dissociated single cells failed to produce comparable behavioral recovery, highlighting the advantages of intact 3D organoid grafts for functional circuit reconstruction.

In summary, neural organoids have demonstrated multi‐level integration with host neural tissue. Structurally, grafts become vascularized and incorporated into the host tissue microenvironment. At the level of neural connectivity, they establish bidirectional synaptic contacts and reconstruct specific long‐range projections. Functionally, transplanted human neurons not only participate in sensory information processing but also modulate host behavior. These insights are supported by rigorous empirical evidence derived from animal models such as immunodeficient mice and neonatal rats, with grafts generated from human pluripotent stem cells, including human cortical organoids. Thus, human brain organoids can be integrated into a living brain anatomically and functionally. This breakthrough may open new avenues for CNS repair.

## 6 Interaction Between Organoids‐BMI Devices and the External Controller

The application of electrical stimulation in the context of Organoid‐BMI devices can be envisioned as a continuum, spanning from in vitro conditioning to long‐term in vivo regulation, while stimulation paradigms are carefully tuned to avoid hyperexcitability or maladaptive activity. The pioneering work from the Li group provides experimental support for this staged framework [90]. At the in vitro stage, electrical stimulation serves as a developmental cue, accelerating neuronal differentiation, network organization, and synchronization, thereby producing more mature and transplant‐ready brain organoids. During the early post‐transplantation phase, stimulation can act as a catalyst for graft‐host integration by modulating glial and immune responses, and promoting directed axonal growth and synapse formation [90]. Moving into the mid‐ to long‐term phase, closed‐loop neuromodulation strategies build upon these foundations, using real‐time decoding and adaptive stimulation to align graft activity with host networks, ultimately enabling individualized functional reconstruction and rehabilitation.

### 6.1 In Vitro Electrical Stimulation Promotes Organoid Maturation and Neural Network Development

**FIGURE 4:**
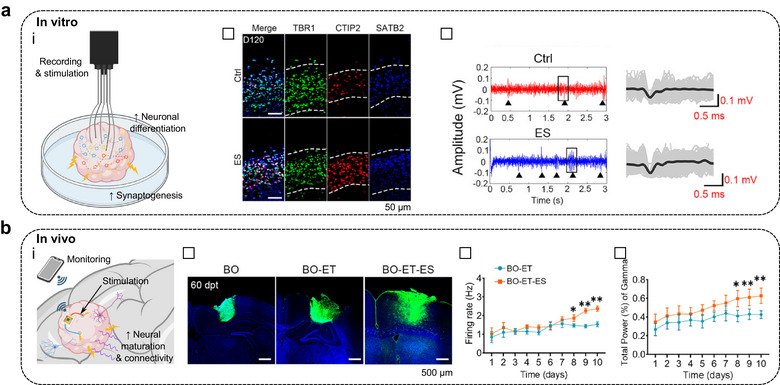
Electrical stimulation enhancing organoid maturation and integration with host circuits. (a) In vitro electrical stimulation promotes cortical maturation. Schematic of the organoid‐electrode culture platform enabling chronic electrical stimulation and electrophysiological recording (i). Immunostaining of day 120 organoids showing enhanced cortical lamination in stimulated organoids (ES), with increased expression of layer‐specific markers TBR1, CTIP2, and SATB2 compared with unstimulated controls (Ctrl) (ii). Representative local field potential recordings and averaged spike waveforms demonstrating increased spike amplitude and more synchronized network activity in ES organoids (iii). (b) In vivo stimulation enhances graft survival and functional integration. Schematic of in vivo transplantation with stimulation applied to grafted organoids (i). Histological analysis shows improved survival and structural integration of stimulated organoids (BO‐ET‐ES) compared with non‐stimulated grafts (BO‐ET) (ii). Quantification of neuronal activity after transplantation reveals significantly increased firing rates and enhanced gamma power in the ES group across 10 days, indicating long‐term circuit engagement and functional plasticity (iii, iv). Adapted from Ref. [90] with permission (2024 Nature Portfolio). Elements created with BioRender.com.

Under in vitro culture conditions, the introduction of electrical stimulation can mimic the bioelectrical activity occurring during brain development and promote the maturation and functional differentiation of brain organoids (Figure 4a,i). Compared with unstimulated controls, electrically stimulated organoids exhibit more distinct cortical lamination and more mature neuronal phenotypes. Electrical stimulation can significantly promote neuronal differentiation and synapse formation, likely by engaging activity‐dependent Ca^2^^+^ signaling pathways such as Ca^2^^+^/CaM/CaMKII and downstream PKA‐CREB activation. Although the precise causal sequence is unclear, accumulating evidence suggests Ca^2^^+^‐mediated signaling as a key bridge between external stimulation and organoid network maturation. Accordingly, careful optimization of stimulation parameters is critical. Comparative experiments applying both low‐ and high‐frequency paradigms indicate that higher‐frequency stimulation (e.g., 250 Hz at ∼400 mV delivered in pulse trains) is associated with greater upregulation of proliferation (Ki67) and neurogenesis markers (DCX) than lower frequencies such as 50 Hz. Importantly, 250 Hz stimulation does not compromise organoid viability or growth, supporting ∼400 mV at 250 Hz as an effective and well‐tolerated condition for promoting organoid development [90]. After a period of repeated stimulation, organoids displayed an increased proportion of neurons and a relative decrease in glial cells, indicating that more progenitors differentiated into mature neurons [90] (Figure 4a,ii). Spontaneous firing frequency and network synchrony were also markedly enhanced, showing more complex electrophysiological activity compared with unstimulated controls [90] (Figure 4a,iii). These findings suggest that electrical stimulation accelerates neural circuit formation and maturation in brain organoids, leading to synchronous network discharges and oscillatory activity resembling those observed during late stages of brain development in vivo [90]. At the molecular level, electrical stimulation alters gene expression in transplanted stem cells, with transcriptomic analyses identifying factors such as stanniocalcin‐2 (STC2) as important mediators of the functional benefits [91]. Taken together, these findings suggest that appropriately designed electrical stimulation protocols, including careful control of electrode configuration, frequency, waveform, and intensity may enhance the anatomical and functional maturation of transplanted neural tissues, including organoids.

### 6.2 Early Post‐Transplantation Stimulation Facilitates Organoid‐Host Integration

Brain organoid transplantation enables structural reconstruction, but early integration remains limited. Moderate electrical stimulation to grafts during the early stages after transplantation facilitates both structural and functional neural integration [90] (Figure 4b,i). In a mouse model of sensory cortex injury, human cortical organoids were transplanted and intermittently stimulated during the early post‐transplant period. Compared with the unstimulated group, stimulated grafts exhibited enhanced structural integration with host tissue (Figure 4b,ii) and showed increased neuronal maturation. Long‐term analyses revealed a broader representation of differentiated cortical subtypes, with increased expression of deep‐layer (CTIP2) and upper‐layer (SATB2) markers [90]. Together, this research suggests that electrical stimulation accelerates the transition of transplanted organoids into organized, functionally relevant cortical tissue. Interface stimulation did not induce marked glial scarring or immune rejection. In the stimulation group, abnormal accumulation of activated microglia (IBA1^+^) or astrocytes (GFAP^+^) was not observed. Most microglia maintained a homeostatic phenotype (P2RY12^+^), and pro‐inflammatory markers (e.g., iNOS) didn't show significant upregulation. This research suggests that appropriately designed stimulation and flexible interfaces may not exacerbate inflammation but rather modulate glial responses to support graft survival and integration. Electrical stimulation enhanced graft integration and modulated the differentiation trajectory of transplanted human iPSC‐derived spinal neural progenitor cells in the injured spinal cord [92]. In one study targeting the visual cortex, when visual stimuli were delivered to the host, transplanted organoids exhibited neural responses synchronized with the host's visual cortex [89]. Specifically, human organoids showed increased multi‐unit activity (MUA), elevated gamma‐band power, and phase‐locking with host low‐frequency rhythms, indicating resonance‐like synchronization (Figure 4b,iii‐iv). Immunohistology further confirmed hybrid synapses between human graft neurons and host neurons. These findings suggest that relevant environmental sensory input may support organoid integration and network activity in a manner analogous to external electrical stimulation, potentially through activity‐dependent modulation of synaptogenesis and axonal growth.

This guiding effect is particularly important in injury settings. In stroke models, electrical stimulation of transplanted cells promoted axonal sprouting and directed growth into perilesional regions [93]. Implantable electro‐cellular hybrid devices were developed and showed that stimulation enhanced graft‐host integration and increased synaptic connectivity between new and existing neurons [94]. Similarly, in a rat stroke model, combining neural progenitor cell transplantation with conductive polymer‐based stimulation led to earlier and more significant sensorimotor recovery compared to either therapy alone. Control groups (cells‐only or electrode‐only) showed limited improvement, while the combination group demonstrated superior neurological scores at 3–4 weeks post‐surgery [91]. Parallel findings have been reported in spinal cord injury models, where combining stem cell transplantation with epidural electrical stimulation produced synergistic effects: acute‐phase grafting improved the local microenvironment, while sustained subacute/chronic stimulation promoted regeneration, together enhancing hindlimb functional recovery [95]. Altogether, these animal models suggest that the introduction of electrical stimulation during the early phase of transplantation can promote both structural and cellular integration.

### 6.3 Closed‐Loop Regulation and Organoid‐Host Interaction in Rehabilitation During Mid‐ to Long‐Term Post‐Transplantation

As the graft establishes basic structural and functional connections with the host, the next step is to achieve individualized functional reconstruction and rehabilitation in the mid‐ to long‐term through closed‐loop neuromodulation. Closed‐loop regulation refers to the bidirectional, real‐time interaction of neural activity via the interfaces. On one hand is to continuously record neural signals from both host and graft; and on the other hand is to adjust stimulation parameters in real time based on these feedback signals, thereby forming an adaptive control loop [90]. Such a bidirectional, closed‐loop organoid‐brain interface framework holds promise for reconstructing specific neural circuits within the host brain, enabling precise disease modulation and functional repair. For example, when patient attempts motor or sensory discrimination tasks, the interface could detect related activity patterns in the brain or graft and trigger appropriate stimulation to amplify or guide correct neural signals, thereby assisting in task completion. Recent exploratory studies have already validated the feasibility of closed‐loop training in vitro. Goal‐directed learning has been demonstrated in cortical brain organoids embedded in a simulated environment (the Cartpole balancing task) within a closed‐loop electrophysiological framework [96]. In this system, organoid firing activity influenced the state of the simulation, while an algorithm, guided by the task objective, dynamically adjusted stimulation patterns applied to the organoid, forming a feedback loop between organoid and environment [96]. With continued training, most organoids demonstrated improved task performance under closed‐loop conditions. For instance, the “Brainoware” platform exhibited enhanced performance in speech recognition tasks after repeated stimulation training [97]. These findings suggest that closed‐loop systems combining sensory feedback and goal‐directed stimulation can induce plastic changes in neural networks, even within ex vivo organoids, thereby enabling a degree of learning and adaptation [38, 97, 98, 99]. By extension, applying similar closed‐loop regulation to transplanted organoid‐host composites in vivo could enable personalized functional retraining tailored to patient‐specific needs [100, 101]. For instance, in individuals with motor deficits caused by cortical injury, a closed‐loop interface could be designed to engage transplanted organoids in motor imagery or actual movement tasks. When the patient attempts to move, the interface records residual brain signals or graft activity, decodes the encoded motor intention in real time, and subsequently delivers patterned electrical stimulation to both the graft and host networks. This encoding strategy amplifies the correct motor control signals. Over the course of training, the system can gradually optimize stimulation parameters, allowing the graft to synchronize more effectively with host motor circuits and ultimately facilitate limb function recovery.

Importantly, the chronic organoid‐host environment is inherently dynamic, and electrode impedance can drift over time due to biocontamination or tissue responses. Furthermore, ongoing transplant maturation and host remodeling alter signal statistics and network coupling. These factors present challenges to system decoding and stimulation, requiring adaptive closed‐loop strategies such as periodic decoder recalibration, impedance‐sensing control, and learning‐based controllers capable of tracking slow bio‐drift. Moreover, robust, long‐term neural encoding and decoding technologies are needed to analyze and process signals from the graft‐host network. In practice, multiple neural biomarkers operating at different temporal scales may serve as control variables in closed‐loop systems, including firing rate and burst statistics (reflecting excitability), oscillatory power and coherence (capturing network synchronization), and cross‐frequency coupling such as phase‐amplitude coupling (PAC), which has been widely implicated in functional integration and plasticity across distributed circuits. Machine learning algorithms can be used to extract and track these biomarkers from closed‐loop recordings. For example, linear decoders or state‐space models can be used for real‐time estimation of firing‐rate‐based variables, and lightweight deep learning models can be employed for capturing nonlinear coupling patterns, thus enabling evaluation of graft‐host integration and adaptive adjustment of stimulation protocols. It is worth noting that long‐term closed‐loop neuromodulation already has successful clinical precedents. For example, closed‐loop deep brain stimulation for Parkinson's disease can automatically adjust stimulation intensity based on the patient's brain state [102, 103, 104] for improving effective symptom control. This provides a valuable reference model for the long‐term application of Organoid‐BMI devices.

In summary, mid‐ to long‐term closed‐loop strategies represent a frontier in the application of organoid‐electrode interfaces for regenerative medicine. By enabling dynamic interaction between graft and host signals, these systems allow individualized rehabilitation, guiding transplanted organoids to exert functional roles in a real‐time, adaptive manner. Such “living biohybrid implants” could emerge as a new generation of implantable neuroprosthetics, designed to replace or reconstruct lost functions of specific brain regions. As relevant technologies and experimental paradigms continue to advance, it is conceivable that innovative studies will soon evaluate closed‐loop Organoid‐BMI devices in animal models—and eventually in clinical settings—by assessing improvements in cognitive or motor task performance and correlating these behavioral gains with activity patterns recorded from the transplanted graft.

## 7 Challenges and Future Perspectives

### 7.1 Organoid Maturity, Heterogeneity, and Standardization Issues

Maturity of organoids remains the first limiting factor for closed‐loop controllability [105]. Most brain organoids generated in vitro display cortical‐like lamination and spontaneous network activity, yet they retain an “early developmental” phenotype with respect to myelination, excitation‐inhibition balance, glial maturation, and rhythm stability [28]. Therefore, culture time is not a reliable criterion for assessing transplant readiness. Inadequately matured grafts often fail to effectively synchronize with host circuits or respond to stimulus‐based training post‐implantation. A more appropriate approach is to conduct a multidimensional assessment of functional maturity before implantation, including: (i) molecular and cellular indicators (layer/area markers, neuronal subtype composition, glial cell maturity); (ii) electrophysiological and network indicators (neural activity, synchronicity, phase‐amplitude coupling); and (iii) connectivity and projection indicators (bidirectional input/output and target specificity). This framework should define the minimum threshold at which a graft is considered “fit for transplant.”

Heterogeneity of organoids further affect their predictability and reproducibility [106, 107]. Cellular lineages and spatial architecture are strongly influenced by the donor genetic background, induction recipes and timing, culture systems (spinner flasks/bioreactors/microfluidics), and size‐dependent metabolic oxygen gradients, producing substantial intra‐ and inter‐batch and even inter‐laboratory variability [108]. Such divergence extends beyond molecular profiles and propagates to in vivo endpoints: graft survival, vascularization kinetics, axonal targeting accuracy, as well as drift in decoding features and stimulation thresholds during closed‐loop operation We must specify a series of systemic mitigation measures: (i) employing region‐specific directed differentiation and controlling shape/size to standardize geometry and metabolic environment; (ii) co‐culturing with endothelial cells, astrocytes, and oligodendrocyte precursor cells to enhance robustness [109]; (iii) implementing process quality control at predetermined milestones (rapid scRNA‐seq sampling and microelectrode arrays, gating); (iv) terminating or rejecting non‐conforming batches; and minimizing operator/environmental variability through automation and statistical process control.

Standardization is critical for advancing Organoid‐BMI devices from research‐grade to clinical‐grade [110]. Beyond compelling histology or short‐term electrophysiology, a clinically oriented Organoid‐BMI devices program requires a fully auditable, traceable, and reproducible pipeline. A pragmatic centerpiece is a “fit for transplant” release checklist encompassing [111] morphology/geometry (size bounds, hypoxia‐necrosis thresholds); composition/maturity (layering and subtype ranges); function (activities baselines, absence of sustained epileptiform activity); safety (low proliferation, no pluripotent remnants, sterility/endotoxin clearance); and interface compatibility (impedance, SNR, charge‐injection capacity, and thermal rise under specified waveforms; accelerated aging/soak durability). In parallel, the field should converge on a minimal critical dataset with harmonized templates for raw single‐cell and electrophysiological data, bidirectional connectivity evidence from tracing/imaging, and standardized post‐implant longitudinal follow‐up (vascularization, inflammation/immune status, survival, and functional endpoints). Such standards are essential to enable cross‐center comparability, secondary analyses, and ultimately regulatory‐grade evidence.

### 7.2 Multimodal Monitoring and Stimulation

Rather than acting as parallel add‐ons, sensing and stimulation modalities in Organoid‐BMI devices play complementary roles within an integrated control architecture across distinct temporal scales and levels of specificity. Electrical stimulation remains the cornerstone of Organoid‐BMI devices research. On the monitoring side, multielectrode arrays, high‐density probes, and flexible implantable electrodes provide high temporal resolution recordings of local field potentials, multi‐unit activity, and network oscillations, supported by relatively mature hardware platforms [112, 113, 114]. In translational settings, the selection of electrode materials needs to be carefully considered, as radiofrequency induction heating under magnetic resonance imaging (MRI) may pose safety risks. On the stimulation side, electrical pulses are highly versatile, capable of directly depolarizing membranes, modulating excitability, and triggering activity‐dependent plasticity. Importantly, electrical stimulation has already been extensively validated in clinical neuromodulation techniques such as deep brain stimulation and spinal cord stimulation, offering translational advantages and a well‐established safety framework. Nevertheless, an exclusive reliance on electrical modalities faces several intrinsic limitations. First, electrical stimulation lacks cell‐type or circuit specificity, often activating heterogeneous populations and fibers of passage, which restricts its precision [115]. Second, the spatial resolution of electrical modulation is inherently constrained: even with microfabricated electrodes, current spread limits the ability to confine modulation to precise microcircuits within organoids or host tissue. Third, electrical recordings capture only fast electrophysiological signals, omitting slower yet equally critical processes such as calcium transients, neurotransmitter release, metabolic dynamics, vascular activity, and systemic biochemical changes that may be reflected in circulating or interstitial biomarkers. Finally, long‐term electrode performance can be compromised by gliosis, impedance drift, and biofouling, which undermines the reliability of closed‐loop control over extended timeframes [116, 117, 118].

Organoid‐BMI devices may be further advanced to address these challenges by integrating multimodal monitoring and stimulation technologies [89, 119]. There are many options and combinations for consideration. For example, optical methods, such as calcium imaging and optogenetic stimulation, may be integrated for neural recording based on their single‐cell resolution and cell‐type specificity. Chemical and molecular sensors can be assembled to directly monitor graft microenvironment by measuring neurotransmitters, growth factors, or inflammatory markers. Metabolic and hemodynamic sensors [120, 121, 122, 123, 124] may be employed to track graft survival by tracking oxygen, glucose, lactate, etc. Furthermore, hybrid approaches, such as microLED‐based neural interfaces [125, 126], may be considered for simultaneous optical and electrical detection of neural activity. In parallel, controlled release systems, such as drug‐loaded hydrogels or microfluidic delivery platforms can be integrated to locally regulate trophic or anti‐inflammatory cues and complement electrical stimulation. Moreover, ultrasound or magnetothermal stimulation may offer contactless deep‐tissue modulation with reduced invasiveness for longer‐term applications [26, 27, 127, 128, 129, 130, 131].

The integration of multimodal strategies could significantly enhance the utility and robustness of Organoid‐BMI devices. Multidimensional readouts that combine electrical, optical, and chemical signals would provide a more comprehensive view of graft‐host integration, capturing structural, functional, and metabolic states simultaneously. In parallel, multimodal stimulation could enable precision control, with cell‐type targeted optical or pharmacological approaches complementing the broad drive of electrical fields. In this layered strategy, global synchronization is achieved through electrical pulses, while fine‐tuned plasticity within defined circuits is modulated via optogenetics or chemogenetics. Moreover, diversified feedback channels would make closed‐loop systems more resilient, maintaining functionality even when one modality deteriorates over time. With the incorporation of advanced AI algorithms, these multimodal data streams could be fused into adaptive frameworks, enabling the identification of individual biomarkers of graft‐host plasticity, dynamic adjustment of stimulation schemes, and the design of personalized rehabilitation strategies. In practice, electrical stimulation could be used to globally synchronize organoid or graft activity, while optogenetic stimulation selectively reinforces specific cell populations or pathways, and chemical or metabolic sensors provide slower feedback to adapt stimulation intensity and timing. In the aging brain, tissue fragility, reduced vascular elasticity, and chronic inflammation are common. Therefore, organoid‐BMI devices must prioritize low‐mechanical‐stress implantation strategies or stimulation modalities driven by adaptive control algorithms to minimize secondary damage. Furthermore, transplanted Organoid‐BMI devices provide a critical platform for preclinical rodent studies, enabling systematic evaluation of long‐term graft‐host integration, safety boundaries, modulation parameters, and functional recovery outcomes in disease‐specific models.

### 7.3 Ethics and Regulation

Organoid‐BMI devices integrate transplantation, brain organoids, information exchange, and behavior control. Once it involves direct connection to the human body, and potentially the control of external devices, it brings us into an unprecedented ethical frontier. First, although the International Society for Stem Cell Research (ISSCR) currently maintains that brain organoids do not yet possess consciousness, it has emphasized the importance of anticipatory governance [132]. As brain organoids increasingly approximate the developmental state of the human brain in terms of electrical activity and structural complexity, they may, in the future, acquire some level of perceptual capacity or even a form of “consciousness.” While the scientific community has yet to clearly define what constitutes “organoid consciousness” [133], this issue touches upon fundamental notions of dignity and therefore cannot be dismissed. Second, because brain organoids are derived from human stem cells, they carry the donor's unique gene expression profile. This raises questions about whether organoids should be considered linked to the donor's personal characteristics, as well as how to define donor identity, intellectual property rights, and privacy [134]. These considerations will determine whether more stringent informed consent procedures and benefit‐sharing mechanisms are required, an issue that has been repeatedly highlighted in ethical debates surrounding brain organoids. Furthermore, Organoid‐BMI devices may access specific reactive patterns within organoids, or even influence the behavior of the host, thus touching on the core of “freedom of thought” and “cognitive privacy.” For this reason, the concept of neurorights is introduced to ensure a fundamental principle of “thoughts are inviolable”. Finally, there is a huge space to further advance current legal and ethical frameworks with the rapid evolution of such technologies. The absence of a comprehensive regulatory framework governing the generation, implantation, and interaction of organoids means that imbalance in any single aspect could introduce significant risks. Without building sufficient trust and transparency between the public and professional communities, applications of Organoid‐BMI devices may face dual resistance from both commercial and ethical fronts.

## Author Contributions

F.G., M.G., J.T., and K.M. conceived the idea. Y.X. wrote the original draft with help from Z. H., C.T., and H. C. Y.Y. supported the schematics. All the authors edited the manuscript.

## Conflicts of Interest

The authors declare no conflict of interest.
